# Prognostic impact of cancer history in patients undergoing transcatheter mitral valve repair

**DOI:** 10.1007/s00392-023-02266-5

**Published:** 2023-08-15

**Authors:** Alev Kalkan, Clemens Metze, Christos Iliadis, Maria I. Körber, Stephan Baldus, Roman Pfister

**Affiliations:** 1 Department III of Internal Medicine, Heart Center, University Hospital of Cologne, Cologne, Germany; 2 Heart Center, University of Cologne, Kerpener Str. 62, 50937 Cologne, North Rhine-Westphalia Germany

**Keywords:** Cancer, Mitral regurgitation, Transcatheter edge-to-edge mitral valve repair, Survival

## Abstract

**Background:**

History of cancer is common in patients undergoing transcatheter mitral valve repair (TMVR).

**Objectives:**

Aim was to examine the impact of cancer history on outcomes after TMVR.

**Methods:**

In patients of a monocentric prospective registry of TMVR history of cancer was retrospectively assessed from records. Associations with 6-week functional outcomes and clinical outcomes during a median follow-up period of 594 days were examined.

**Results:**

Of 661 patients (mean age 79 years; age-range 37–101 years; 56.1% men), 21.6% had a history of cancer with active disease in 4.1%. Compared with non-cancer patients, cancer patients had a similar procedural success rate (reduction of mitral regurgitation to grade 2 or lower 91.6% vs. 88%; p = 0.517) and similar relevant improvement in 6-min walking distance, NYHA class, Minnesota Living with Heart Failure Questionnaire score and Short Form 36 scores. 1-year survival (83% vs. 82%; p = 0.813) and 1-year survival free of heart failure decompensation (75% vs. 76%; p = 0.871) were comparable between cancer and non-cancer patients. Patients with an active cancer disease showed significantly higher mortality compared with patients having a history of cancer (hazard ratio 2.05 [95% CI 1.11–3.82; p = 0.023]) but similar mortality at landmark analysis of 1 year.

**Conclusion:**

TMVR can be performed with equal efficacy in patients with and without cancer and symptomatic mitral regurgitation. Cancer patients show comparable clinical outcome and short-term functional improvement as non-cancer patients. However, longterm mortality was increased in patients with active cancer underlining the importance of patient selection within the heart-team evaluation.

**Graphical abstract:**

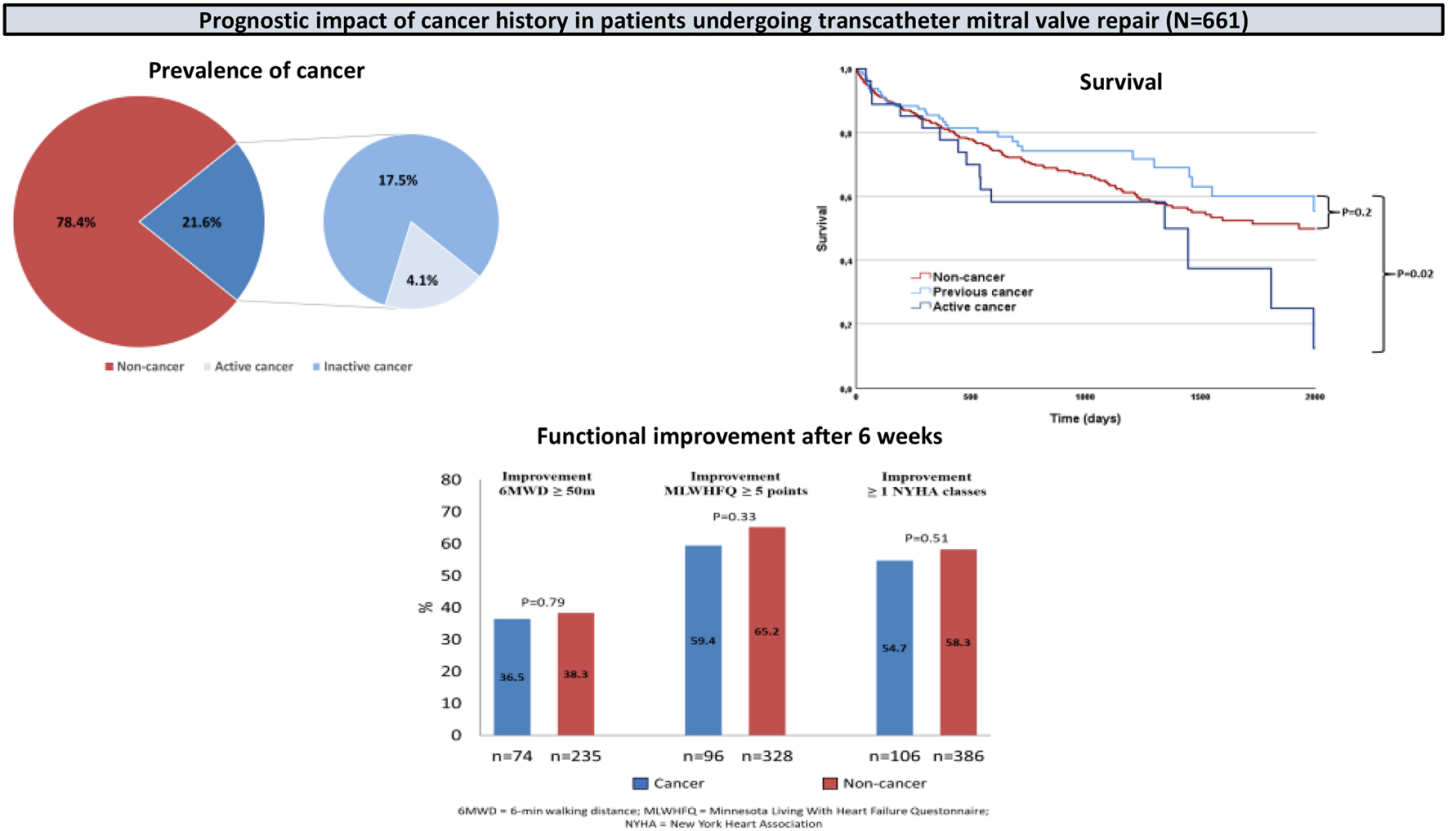

**Supplementary Information:**

The online version contains supplementary material available at 10.1007/s00392-023-02266-5.

## Introduction

Transcatheter mitral valve repair (TMVR) using edge-to-edge technique has evolved as standard of care in symptomatic patients with severe mitral regurgitation who exhibit an increased risk for a surgical procedure [1]. Overall safety and efficacy of TMVR have been confirmed in several studies [2, 3]. However, many of the cardiac and extracardiac comorbidities which contribute to surgical risk and subsequently the decision for a transcatheter therapy approach might specifically impact procedural and procedure independent patient outcomes and the clinical potential of symptomatic and functional benefit after TMVR. Hence, efficacy and safety of TMVR in the background of such common comorbidities need particular attention and evaluation.

Considering demographic changes and increased expectancy of life, incidence and prevalence of oncological diseases rise [4]. In 2019, there were an estimated 23.6 million new cancer cases worldwide and 10.0 million cancer-related deaths [5]. At the same time, life expectancy in cancer patients continues to increase because of both advances in early detection and novel treatment options [4, 6]. Besides the risk of cancer relapse, secondary tumors or ongoing cancer therapy patients with a history of cancer also have an increased risk of impairments in multiple organ systems which can adversely impact functionality. Furthermore, cancer patients are at increased risk of experiencing cardiovascular complications, which can contribute to total morbidity and mortality in these patients and attenuate the benefit of TMVR [6, 7].

Due to the rising incidence of cancer, these patients are no longer rare in studies with TMVR [8]. Nevertheless, dedicated analysis on the impact of cancer not only on clinical outcome after TMVR but also on functional outcomes are lacking. The aim of this study was to examine the impact of cancer on clinical outcomes in patients undergoing TMVR in a large referral center.

## Methods

### Study population

Based on our prospectively captured database, all consecutive patients who were admitted for TMVR to the Heart Centre of the University of Cologne between December 2012 and December 2019 were eligible for this study. Exclusion criteria were missing informed consent or age < 18. All patients had symptomatic (3rd to 4th degree according to current guidelines [9]) mitral valve regurgitation as documented by pre-procedural echocardiography. All patients were discussed by an interdisciplinary heart team consisting of cardiac surgeons, interventional cardiologists, imaging specialists and cardiac anesthesiologists and a decision on transcatheter therapy was made according to current guidelines based on objective risk score, individual patient characteristics, left-ventricular function and mitral valve morphology [9]. In patients with active cancer, cardio-oncology consultation was obtained for estimated life expectancy and intended cancer treatment.

Procedures were performed under general anesthesia. All interventional cardiologists performing TMVR had a minimum experience of 100 procedures as well as a 5-year experience in interventional cardiology [10].

Written informed consent was acquired from all patients. The study was approved by the local ethics committee of the University of Cologne [13–15] and was conducted in accordance with the Declaration of Helsinki.

### Baseline assessment

Patient data was extracted from the electronic patient record. On the day before the intervention, the patients were admitted to the hospital. At this point every patient received a prespecified standardized assessment. Demographic parameters such as age, height, weight, blood pressure and heart rate were recorded. Relevant pre-existing conditions such as a history of cancer, arterial hypertension, diabetes mellitus, atrial fibrillation were surveyed.

Echocardiography was performed to assess the severity and mechanism of the mitral regurgitation such as ejection fraction (Simpson, %), left ventricular end-diastolic diameter (abbr.: LVEDD, mm) and LVESD (mm) by a senior cardiologist before TMVR as well as at discharge after TMVR. Furthermore, the following questionnaires were collected by a trained medical student before the procedure: Short Form 36/Short Form 12 (SF36 physical and mental component score [PCS and MCS]), Minnesota living with heart failure (MLWHFQ), New York Heart Association (NYHA). The patients also completed a 6-minute walk test (6MWT). Detailed assessment with SF36, MLWHFQ and 6MWT were only performed between May 2014 and October 2017.

### Distinction between cancer and non-cancer group

Patients were divided into a cancer and non-cancer group. The cancer-group was defined as a history of a malignant disease or a currently active malignant disease. Moreover, the malignancy was divided by primary cancer location into eight subgroups (pulmonary, myeloid- and lymphatic system, colorectal and urogenital cancer, gynecological, cerebral and others). History and status of cancer was extracted from patient records. In case of active cancer we defined ongoing cancer disease with or without treatment. Subgroup analyses were performed for cancer patients with history of chest irradiation and patients with cancer localization other than urogenital, assuming that these patients might have higher cardiotoxic risk due to their cancer therapy.

### Follow-up

Follow-up was performed after 6 weeks in our outpatient clinic, where patients appeared in person. If the patient did not present personally, a telephone survey was carried out. In addition to basic examinations such as electrocardiogram, transthoracic echocardiography and laboratory tests, standard follow-up also included the collection of questionnaires for the baseline assessment (Minnesota Living With Heart Failure Questionnaire and Short-Form 12) and 6 Minute Walk Test. Long-term follow-up was assessed by telephone with the patient, the treating physician, relatives or obtaining discharge letters.

The following endpoints were evaluated based on the endpoint definition of the Mitral Valve Academic Research Consortium: All-cause mortality, hospitalization for heart failure decompensation, access and vascular complications (major access related vascular complication or vascular surgery at access site). Moreover, we evaluated the procedural success defined as reduction of mitral valve insufficiency to ≤ 2.

### Statistical analysis

Statistical analysis was conducted using SPSS-Software (Version 28.0). Patients with history of cancer were compared with those without cancer. Descriptive parameters were presented as mean ± SD or as median with interquartile range (IQR). In case of non-normal distribution, the differences in both groups were analyzed by the Mann–Whitney U test. In case of normally distributed values, Student’s T-test was used. Kaplan–Meier analysis was used to show survival and event-free survival, and a Log-Rank test was outperformed to examine statistical significance between groups. Statistical significance was defined as p-value < 0.05.

## Results

### Baseline characteristics

**Fig. 1:**
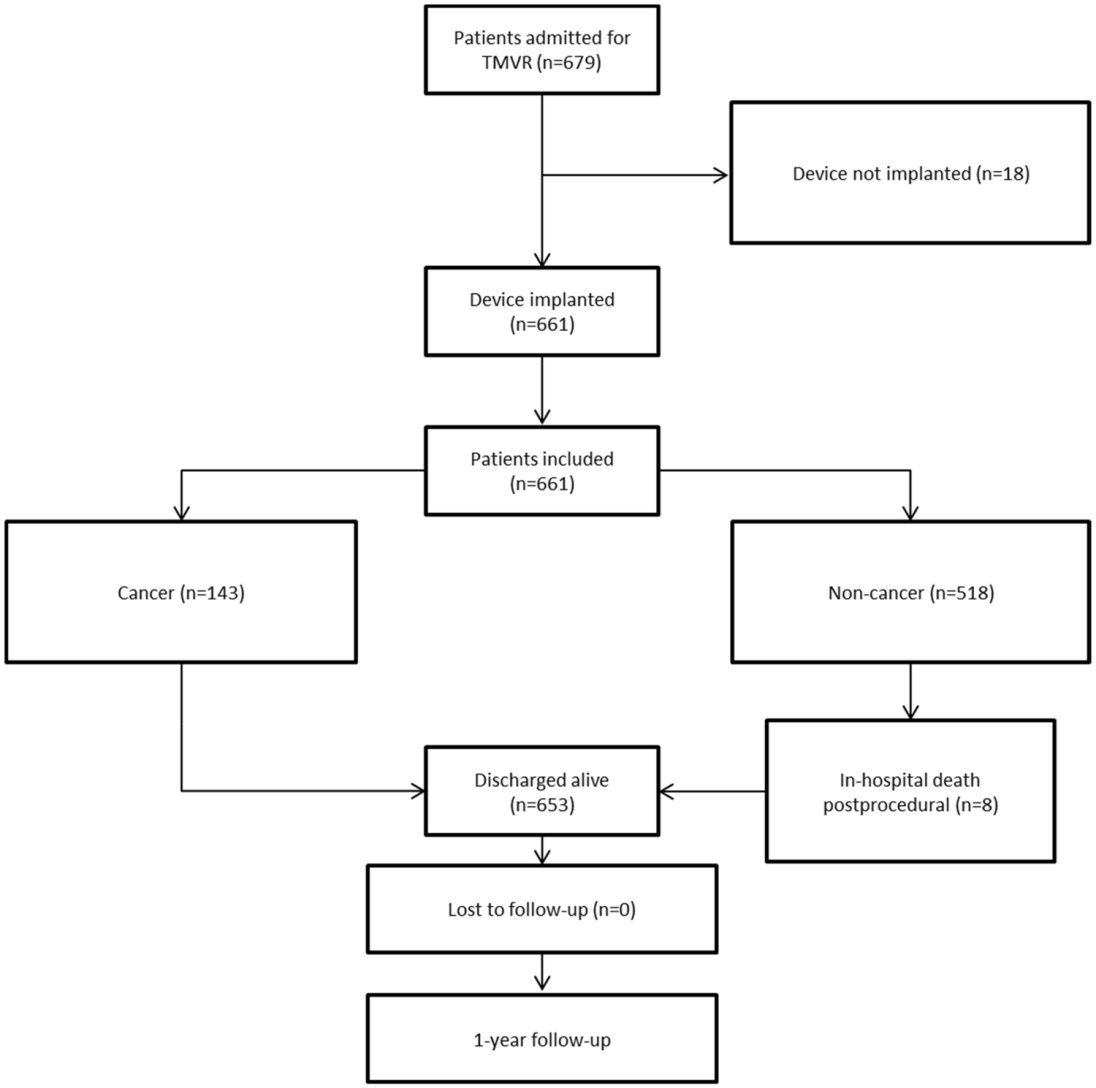
Inclusion diagram

679 patients were admitted for first edge-to-edge TMVR at our institution during the study period. In 18 patients (2.7%, 2 cancer patients and 16 non-cancer patients) starting the TMVR procedure, the device could not be implanted, leaving 661 patients (mean age: 79 years [IQR 73; 83]; 56.1% male) for this analysis (Fig. 1). Underlying pathology of MR was primary/degenerative in 39.8%, secondary/functional in 54.3%, or combined primary and secondary in 5.9%.

143 patients (21.6%) had a history of cancer with the initial first diagnosis of cancer made a median of 72 months (IQR 24; 144) before TMVR. 116 patients (81.1%) had prior history of cancer and 27 patients (18.9%) had an active cancer disease at the time of TMVR, with 10 patients (7%) currently receiving cancer therapy. Patients with an active cancer disease received significantly less often cancer surgery and numerically more often radiation therapy (Table 1).

**Table 1:** Status of cancer and cancer treatment, n (%)

	Total cancer patients (n = 143)	Active cancer (n = 27)	History of cancer (n = 116)	p-Value
Chemotherapy	12 (8.4)	4 (14.8)	8 (6.9)	0.24
Cardiotoxic chemotherapy	7 (4.9)	2 (7.4)	5 (4.3)	0.42
Radiation	22 (15.4)	8 (29.6)	14 (12.1)	0.063
Chest irradiation	8 (5.6)	7 (25.9)	1 (0.9)	0.29
Cancer surgery	85 (59.4)	10 (37)	75 (64.7)	0.009
No therapy/watch and wait	3 (2.1)	2 (7.4)	1 (0.9)	0.091

**Fig. 2:**
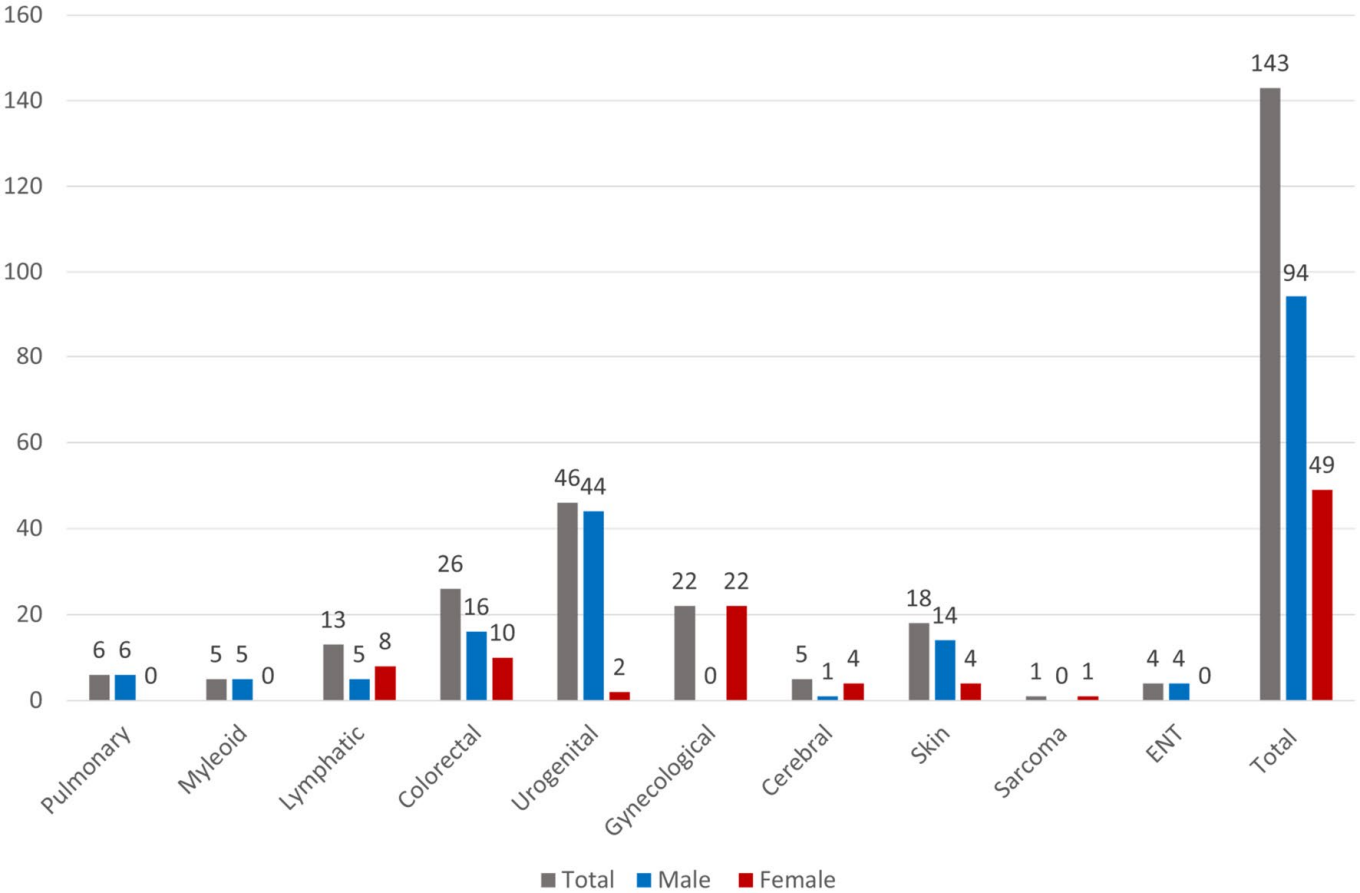
Type of cancer

Urogenital (32.2%), colorectal (18.2%), and gynecological (15.4%) were the most prevalent cancer types in total. The most prevalent cancer types in male patients were urogenital (46.8%) and colorectal (17%), whereas the most prevalent cancer types in female patients were gynecological (44.9%) and colorectal (20.4%) (Fig. 2).

At baseline, cancer and non-cancer patients were similar regarding age, BMI, pathology of MR, structural cardiac disease, logistic EuroSCORE and ejection fraction. There was also no difference regarding functional status including NT-pro-BNP levels, NYHA-class, 6MWD, SF36 MCS and PCS and MLWHFQ score. Cancer patients showed a higher rate of male gender (65.7% vs. 53.5% male; p = 0.01) (Table 2).

**Table 2:** Baseline characteristics of the total study population and by cancer status

	Total (n = 661)	Cancer (n = 143)	Non-cancer (n = 518)	p-Value
Gender, male n (%)	371 (56.1)	94 (65.7)	277 (53.5)	0.01
Age, years	79 (73; 83)	80 (75; 84)	79 (79; 83)	0.122
BMI, kg/m^2^	24.7 (22.5; 28)	25.1 (22.2; 27.9)	24.7 (22.5; 28.1)	0.715
Cause of MR, n (%)				0.71
Functional MR	322 (54.3)	67 (51.1)	255 (55.2)	
Degenerative MR	236 (39.8)	56 (42.7)	180 (39)	
Combined pathology	35 (5.9)	8 (6.1)	27 (5.8)	
Structural cardiac disease, n (%)	493/661			0.842
Ischemic cardiomyopathy	256 (51.9)	55 (50.9)	201 (52.2)	
Dilated cardiomyopathy	180 (36.5)	38 (35.2)	142 (36.9)	
None	57 (11.6)	15 (13.9)	42 (10.9)	
Log. Euroscore, %	11 (6.2; 21.3)	10.8 (6.8; 23.6)	11 (6.1; 21.2)	0.72
LVEF, n (%)				0.31
> 50%	238 (42.4)	59 (48)	179 (40.9)	
30–50%	186 (33.2)	39 (31.7)	147 (33.6)	
< 30%	137 (24.4)	25 (20.3)	112 (25.6)	
Diabetes mellitus, n (%)	174 (26.3)	40 (28)	134 (25.9)	0.8
Previous stroke, n (%)	94 (14.2)	20 (14)	74 (14.3)	0.931
Previous myocardial infarction, n (%)	173 (26.2)	27 (18.9)	146 (28.2)	0.066
Coronary artery disease, n (%)	383 (58.6)	84 (58.7)	299 (57.7)	0.923
Previous cardiac surgery, n (%)	203 (30.7)	39 (27.3)	164 (31.7)	0.273
Previous mitral valve surgery, n (%)	19 (2.9)	2 (1.4)	17 (3.3)	0.392
Atrial fibrillation, n (%)	439 (67.3)	105 (73.4)	334 (64.5)	0.047
LV enddiastolic diameter, mm	55 (49; 62)	53.5 (48; 59.3)	56 (49; 62.3)	0.037
LA area, cm^2^		26 (23; 36.7)	30 (26; 34.2)	0.054
Tricuspid regurgitation, n (%)				0.27
Grade 0/1	148 (42.4)	31 (37.3)	117 (44)	
Grade 2	92 (26.4)	27 (32.5)	65 (24.4)	
Grade 3/4	109 (31.2)	25 (30.1)	84 (31.5)	
eGFR, ml/min	41.6 (30.1; 57.6)	38.3 (29.6; 52.1)	42.6 (30.5; 58.6)	0.118
NT-pro-BNP, ng/l	2986 (1473; 6396)	3302 (1508; 6444)	2989 (1470; 6403)	0.7
NYHA class, n (%)				0.46
NYHA 1–2	63 (9.5)	17 (12.1)	46 (9)	
NYHA 3–4	588 (89)	124 (87.9)	464 (91)	
6MWD, m	249 (132)	253 (135)	248 (131)	0.733
MLWHFQ score	33 (22; 47)	32 (21; 45)	33 (22; 49)	0.39
SF-36				
Physical component score, 0–100	35.4 (8.8)	34.5 (8.3)	35.7 (9)	0.31
Mental component score, 0–100	52.5 (44.3; 58.6)	53.3 (45.9; 60.1)	51.9 (44.2; 58.3)	0.446

Mean (standard deviation), median (interquartile range), or frequency (percentage) as appropriate

*BMI* body mass index, *MR* mitral regurgitation, *LVEF* left-ventricular ejection fraction, *eGFR* estimated glomerular filtration rate, *6MWD* 6 min walking distance, *MLWHFQ* Minnesota Living with Heart Failure Questionnaire, *SF* Short Form

p-Value for comparison of cancer and non-cancer patients

Compared to patients with a history of prior cancer, patients with an active cancer disease were younger, had a higher BMI and a larger left-ventricular end-diastolic diameter, and showed a worse baseline MLWHFQ score (Table 3).

**Table3:** Baseline characteristics of the study patients by activity of cancer

	Active cancer (n = 27)	History of cancer (n = 116)	p-Value
Gender, male n (%)	22 (81.5)	72 (62.1)	0.072
Age, years	77 (68; 82)	80 (76; 84)	0.012
BMI, kg/m^2^	26.4 (23.4; 29.4)	24.3 (22.1; 27.4)	0.049
Cause of MR, n (%)			0.054
Functional MR	18 (72)	49 (46.2)	
Degenerative MR	7 (28)	49 (46.2)	
Combined pathology	0	8 (6.9)	
Structural cardiac disease, n (%)			0.89
Ischemic cardiomyopathy	11 (55)	44 (50)	
Dilated cardiomyopathy	6 (30)	32 (36.4)	
None	3 (15)	12 (13.6)	
Log. Euroscore, %	10.3 (6.9; 17.1)	11 (6.5; 24.6)	0.642
LVEF, n (%)			0.88
> 50%	11 (44)	48 (49)	
30–50%	8 (32)	31 (31.6)	
< 30%	6 (24)	19 (19.4)	
Diabetes mellitus, n (%)	12 (44.4)	28 (24.1)	0.055
Previous stroke, n (%)	4 (14.8)	16 (13.8)	0.76
Previous myocardial infarction, n (%)	8 (29.6)	19 (16.4)	0.174
Coronary artery disease, n (%)	16 (59.3)	68 (58.6)	0.99
Previous cardiac surgery, n (%)	7 (25.9)	32 (27.6)	0.82
Previous mitral valve surgery, n (%)	0	2 (1.7)	1
Atrial fibrillation, n (%)	18 (66.7)	87 (75)	0.47
LV enddiastolic diameter, mm	58 (53; 65)	52 (47; 58)	0.022
LA area, cm^2^	30 (26.8; 50.1)	25 (22.6; 36.2)	1
Tricuspid regurgitation, n (%)			0.51
Grade 0/1	6 (31.6)	25 (39)	
Grade 2	5 (26.3)	22 (34.4)	
Grade 3/4	8 (42.1)	17 (26.6)	
eGFR, ml/min	37.6 (26.6; 63)	40.5 (29.6; 51.2)	0.741
NT-pro-BNP, ng/l	5186 (2485; 6684)	3153 (1399; 6032)	0.082
NYHA class, n (%)			1
NYHA 1–2	3 (11.1)	14 (12.1)	
NYHA 3–4	24 (88.9)	100 (86.2)	
6MWD, m	257 (136)	253 (136)	
MLWHFQ score	40 (30; 56)	29 (20; 43)	0.039
SF-36			
Physical component score, 0–100	34.5 (7.7)	34.5 (8.5)	0.84
Mental component score, 0–100	52.6 (39.5; 61.8)	53.4 (48.2; 59.6)	0.691

Mean (standard deviation), median (interquartile range), or frequency (percentage) as appropriate

*BMI* body mass index, *MR* mitral regurgitation, *LVEF* left-ventricular ejection fraction, *eGFR* estimated glomerular filtration rate, *6MWD* 6 min walking distance, *MLWHFQ* Minnesota Living with Heart Failure Questionnaire, *SF* Short Form

p-Value for comparison of cancer and non-cancer patients

### Procedural results and clinical outcome

Procedural success with reduction of MR to grade 2 or lower was similar in cancer and non-cancer patients (91.6% vs. 88%; p = 0.517). Procedural success was also similar in patients with a history of chest irradiation (87.5%). There was no significant difference regarding procedural complications, or the length of hospital stay (7 days [IQR 5; 10] vs. 7 days [IQR 5; 11]; p = 0.161) (Table 4). Median follow up time was 594 days (IQR 361; 1056) with 216 patients (32.7%) deceased since baseline assessment and 102 patients (15.4%) hospitalized for heart failure decompensation. Cancer and non-cancer patients showed a similar median survival time (1993 days vs. 1929 days; p = 0.623) and a similar median survival time free of heart failure hospitalization (1441 days vs. 1318 days; p = 0.858).

**Table 4:** Procedural results by cancer status

	Cancer (n = 143)	Non-cancer (n = 518)	p-Value
Procedural success with pre-discharge reduction MR to ≤ 2, n (%)	131 (91.6)	456 (88)	0.517
Pre-discharge LVEF, n (%)			0.387
> 50%	38 (42.7)	109 (36)	
30–50%	25 (28.1)	107 (35.3)	
< 30%	26 (29.2)	87 (28.7)	
Pre-discharge LV enddiastolic diameter, mm	54.5 (49; 60.8)	56 (48.8; 65.3)	0.166
Pre-discharge LA area, cm^2^	31.1 (24.5; 36.5)	29 (24; 34.5)	0.194
Pre-discharge tricuspid regurgitation, n (%)			0.656
Grade 0/1	23 (35.9)	68 (36.4)	
Grade 2	22 (34.4)	76 (40.6)	
Grade 3/4	18 (29.7)	43 (23)	
Major cardiac structural complications related to access			
Pericardial effusion necessitation pericardiocentesis, n (%)	0	2 (0.4)	1
Major access-related vascular complication			
Vascular surgery at access site, n (%)	1 (0.7)	6 (1.2)	1
Length of hospital stay, days	7 (5;10)	7 (5;11)	0.161

Median (interquartile range), or frequency (percentage) as appropriate

p-Value for comparison of cancer and non-cancer patients

**Fig. 3:**
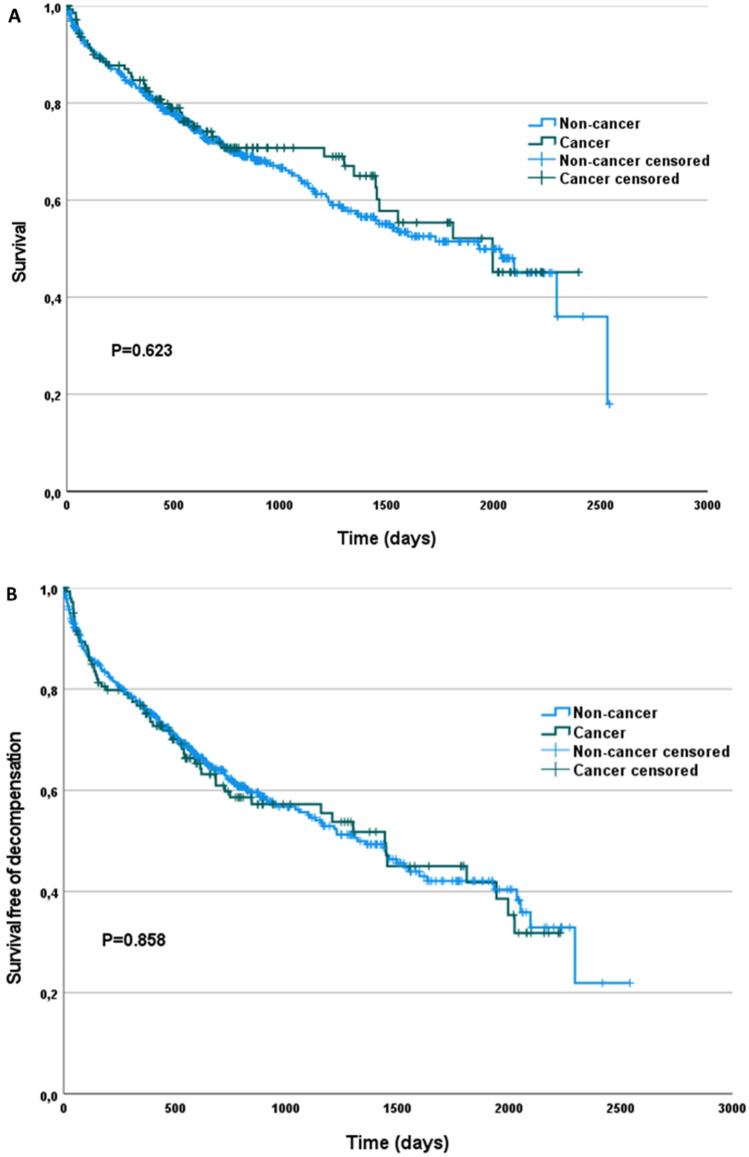
Kaplan–Meier survival plot for mortality (**A**) and mortality or heart failure hospitalisation (**B**) by history of cancer

**Fig. 4:**
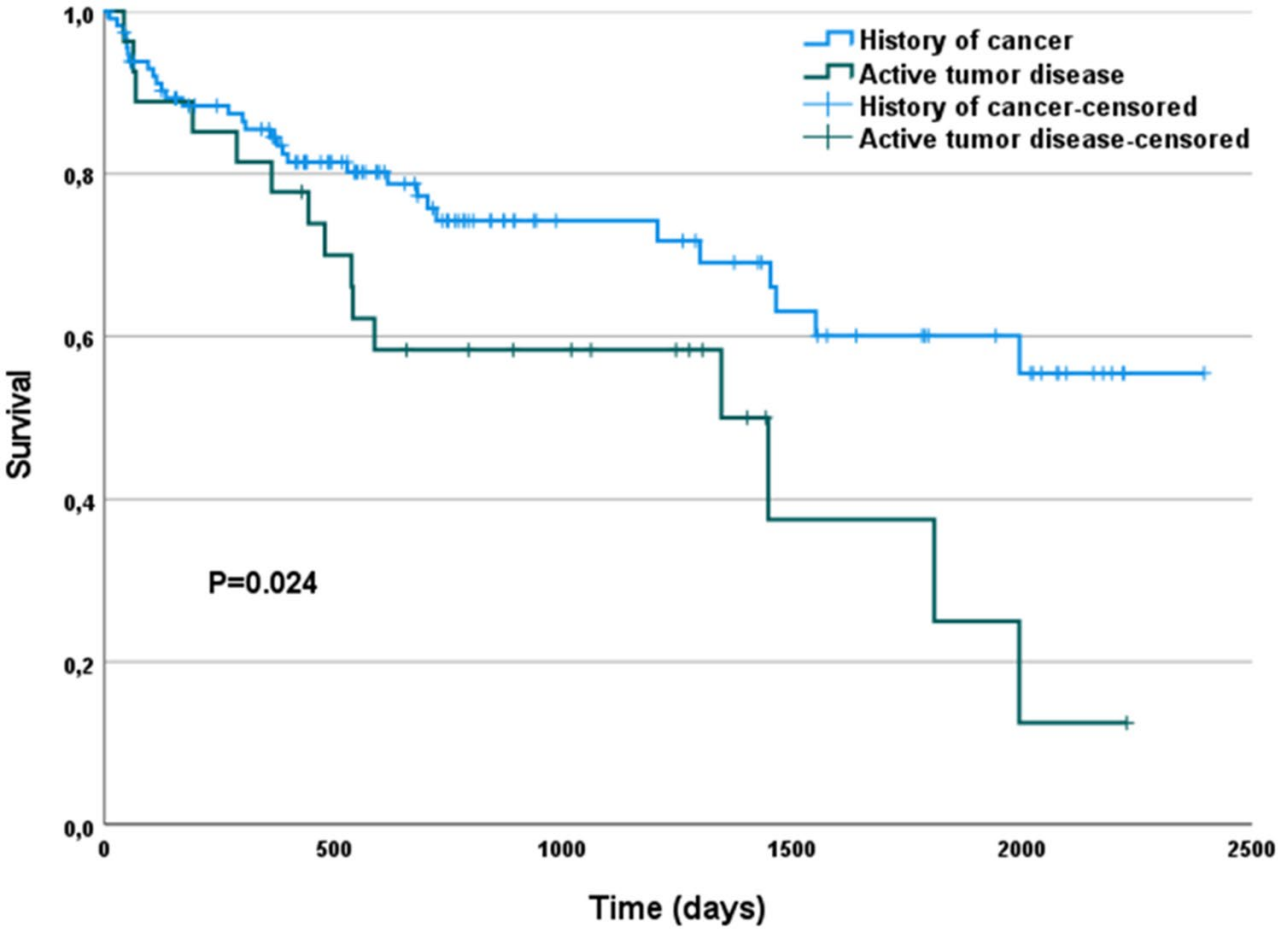
Kaplan–Meier survival plot for mortality by activity of cancer

1-year survival (Kaplan–Meier estimated probability) was 0.83 in cancer and 0.82 in non-cancer patients (log-rank p = 0.813) (Fig. 3A). 1-year survival free of heart failure hospitalization (Kaplan–Meier estimated probability) was 0.75 in cancer and 0.76 in non-cancer patients (log-rank p = 0.871) (Fig. 3B). Patients with an active cancer disease showed significantly lower survival compared with patients having a history of cancer (Fig. 4). The hazard ratio (HR) of death associated with active compared to previous cancer disease was 2.05 (95% CI 1.11–3.82; p = 0.023).

There was no significant difference regarding 1-year survival (81 vs. 85%; p = 0.315) between patients with active and previous cancer.

The type of cancer treatment regarding the two most prevalent types of cancer surgery and irradiation did not influence 1-year survival and 1-year survival free of heart failure rehospitalization. Also, 1-year survival (Kaplan–Meier estimated probability) did not differ significantly between urogenital type of cancer and other type of cancer (76 vs. 86%; log-rank p = 0.37).

### Outcome of functional status

The rate of patients with detailed functional assessment at 6-week follow-up visit defined by availability of MLHWFQ data was 428 (64.8%), due to missed assessment during the visit or lack of a personal appointment due to long distance to our center. Available functional assessment was similar in cancer (67.1%) compared to non-cancer patients (64.1%; p = 0.553).

**Fig. 5:**
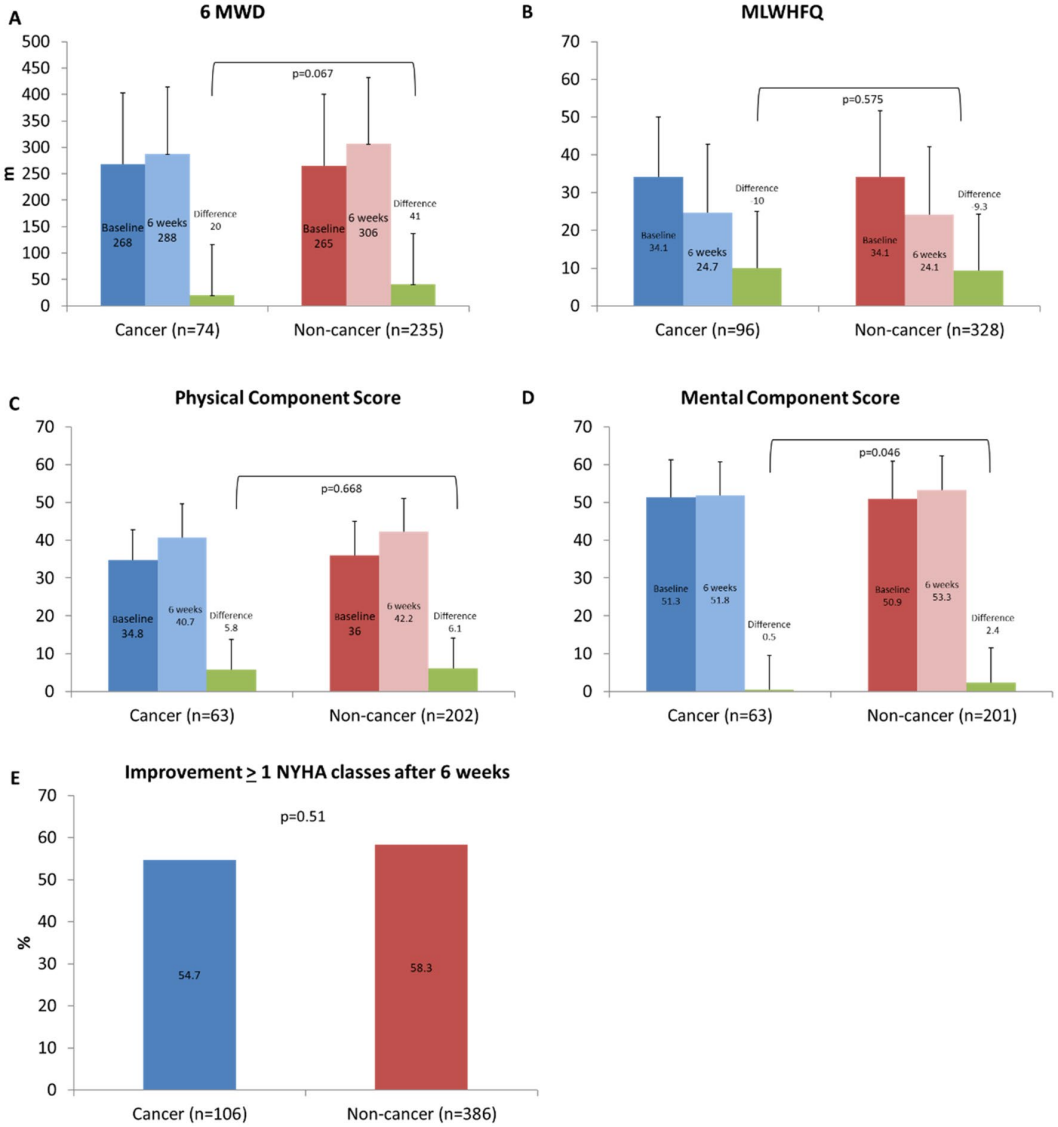
Changes in functional parameters from baseline to 6 weeks. Depicted are baseline, 6-week, and absolute changes (delta) in 6 min walking distance (6 MWD) (**A**), Minnesota Living with Heart Failure Questionnaire (MLWHFQ) score (range 0–105; higher scores indicate worse quality of life) (**B**), physical component (**C**) and mental component (**D**) scores (range 0–100; higher scores indicate better physical and mental status), and improvement of ≥ 1 New York Heart Association functional class at 6 weeks (**E**). p-Value for comparison of absolute changes between cancer and non-cancer patients

At 6-week follow-up functional parameters NYHA class, 6 MWD, MLWHFQ score and SF36 MCS and PCS improved both in cancer and non-cancer patients (Table 5). The magnitude of improvement and the rate of patients with clinically relevant improvement did not differ significantly between both groups (Fig. 5). For instance, the rate of patients with an improvement in MLWHFQ score of 5 points or more was 59.4% in cancer patients and 65.2% in patients without cancer. There was no relevant difference between cancer patients having an active disease compared to those having a history of cancer, between those with and without previous chest irradiation and between those with an urogenital and other cancer type regarding clinically relevant improvement (Suppl. Figures 1 and 2).

**Table 5:** Outcome parameters by cancer status

	Cancer (n = 143)	Non-cancer (n = 518)	p-Value
Death, n (%)	46 (32.3)	170 (32.8)	0.92
Periprocedural (inhospital or < 30 days)	2 (1.4)	19 (3.7	0.287
Post discharge/> 30 days	44 (30.8)	151 (29.2)	0.87
< 1 year	26 (18.2)	91 (17.6)	0.901
Cardiovascular cause, n (% of death)	9 (19.6)	55 (32.4)	0.391
Decompensation with rehospitalisation, n (%)	23 (16.1)	79 (15.3)	0.896
Periprocedural (< 30 days)	1 (0.8)	11 (2.1)	0.323
> 30 days	22 (15.4)	68 (13.1)	0.31
< 1 year	12 (8.4)	52 (10)	0.634
Death or decompensation with rehospitalisation, n (%)	62 (43.4)	211 (40.7)	0.632
< 1 year	36 (25.2)	131 (25.3)	1
Functional outcomes (6 weeks)			
Change 6MWD, m	20 (99)	41 (102)	0.124
Improvement 6MWD ≥ 50 m (n/N, %)	27/74 (36.5)	90/235 (38.3)	0.786
Improvement PCS	5.8 (8)	6.1 (8.6)	0.8
Improvement PCS ≥ 2.5 points (n/N, %)	45/63 (71.4)	141/202 (69.8)	0.469
Improvement MCS	0.5 (8.6)	2.4 (9.6)	0.156
Improvement MCS ≥ 2.5 points (n/N, %)	25/63 (39.7)	104/201 (51.7)	0.063
Improvement MLWHFQ	9.3 (14)	10 (15)	0.729
Improvement MLWHFQ ≥ 5 points (n/N, %)	57/96 (59.4)	214/328 (65.2)	0.334

*6MWD* 6 min walking distance, *MLWHFQ* Minnesota Living with Heart Failure Questionnaire, *SF* Short Form, *PCS* physical component score, *MCS* mental component score

p-Value for comparison of cancer and non-cancer patients

## Discussion

Prevalence of both cardiovascular and cancer diseases increases with age [11]. At the same time, prevalence of mitral valve regurgitation is estimated at 1–2% in the general population and increases significantly in the elderly population, with prevalence of more than 10% in higher age groups. Based on our data from a large TMVR referral center almost one in five patients had a history of cancer and 7% of patients had active cancer disease. Procedural efficacy and safety as well as midterm mortality were not significantly different between patients with and without cancer. Nonetheless, patients with active cancer disease had a significantly increased midterm mortality beyond 1 year after TMVR. Cancer patients showed a similar improvement in NYHA class after TMVR compared with non-cancer patients. Also, the rate of clinically relevant improvement of SF-36 physical component score and MLWHFQ score were not significantly different in cancer compared with non-cancer patients, no matter whether cancer was active or not.

Few studies assessed cancer frequency in TMVR patients so far. In the German Transcatheter Mitral Valve Interventions registry rate of baseline malignancy was 9.3%. However, it is unclear whether any history of cancer was considered or only non-curative cases. Other monocentric studies reported cancer frequency between 11 and 18% [12, 13]. Hence, history of cancer is a substantial comorbidity in TMVR patients which justifies special consideration.

The impact of cancer on patients undergoing cardiovascular interventions has been studied in several settings with inconsistent results. In context of percutaneous coronary interventions for acute coronary syndrome (ACS), patients with a history of cancer had increased rates of in-hospital and 1-year all-cause death and cardiac death [14]. Similar findings could be observed by Velders et al. [15] in patients with ST elevation myocardial infarction. However, another study performed by Hess et al. [16] did not show a significant difference regarding cardiovascular events between the two groups after percutaneous coronary intervention. In the context of transcatheter aortic valve implantation, Tabata et al. [17] found that a history of cancer was a significant predictor of 5-year mortality. Another large nationwide US study reported a significant association with mortality only for lung cancer but not for breast and colorectal cancers [18] whereas colon cancer was a potent risk factor for periprocedural bleeding [19]. Taken together, the expected impact of cancer on postprocedural survival will strongly depend on patient selection with respect to type and stage of cancer, but also procedural characteristics. This is particularly relevant for the interpretation of studies on elective procedures where patients with high-risk cancer can be rejected in the first place.

Guidelines generally recommend interventional cardiovascular therapies only if patient`s life expectancy with acceptable quality is more than 1 year [9]. This important criterion has been also considered in our heart-team decisions for patients with cancer. Hence, even in patients with active cancer 1-year survival was high and not different from patients without cancer. Albeit we do not have data available about patients rejected for TMVR at our center, high-risk cancer types like lung cancer are underrepresented in our treated patients, suggesting a patient preselection by cancer related prognosis. In fact, supposedly low-risk cancer types with urogenital localization showed similar 1-year survival as the other cancer types.

Study results regarding the impact of cancer on postprocedural survival are rare in TMVR [12, 13]. In a monocentric study Oner et al. [13] showed a more than doubled mortality risk associated with previous history of cancer with an estimated 1-year mortality of 56%. The enrollment of this study was in early years of TMVR where patients were required to have overall higher risk profiles. Notable for this study is also the small patient cohort of 19 cancer patients and a significantly lower technical and device success, longer hospitalization and an increased overall risk profile in the cancer patients, all of which have been demonstrated to be independent predictors of increased mortality [13]. Tabata et al. [12] demonstrated in a cohort of 446 TMVR patients that cancer patients also exhibit a worse prognosis with a more than doubled mortality risk and an estimated 1-year mortality rate of 20%. The risk increase remained significant after adjustment for other risk factors. Of note, the 1-year mortality of cancer patients in this study was similar to our cancer patients, but the non-cancer patients showed a better outcome than our patients. In line with this, the distribution of cancer types and the rate of active cancer patients was comparable across both cohorts. Despite the overall comorbidity being also comparable, our cohort had a higher rate of secondary mitral regurgitation and lower left-ventricular ejection fraction which can explain the overall higher mortality. Albeit this is an indirect comparison with respective limitations, the 1-year mortality in the Society of Thoracic Surgeons/American College of Cardiology Transcatheter Valve Therapy Registry comprising more than 33,000 TMVR procedures was 22%, suggesting that our cohort was rather representative with respect to mortality [20], and the differences in mortality between cancer and non-cancer patients observed by Tabata et al. might be attributable to distinct patient selection.

A major finding of our study is that the benefit in symptoms, physical capacity and quality of life is similar in cancer and non-cancer patients, even in patients with active cancer. The main treatment aim of TMVR is symptomatic benefit in the majority of patients. This is of major relevance in the context of cancer, since a body of evidence shows a general acceleration of functional decline and subsequent impairment in quality of life associated with cancer survivorship [21]. For instance, a particular decline in physical function has been reported in patients with prostate, breast, bladder, colorectal, and kidney cancer [22]. This phenomenon is likely to be multifactorial with tumor and tumor therapy related factors playing an important role [23] and is seen on long-term follow-up in tumor survivors. Jefford et al. [24] demonstrated that survivors generally reported poorer quality of life compared to the general population after 5 years post-diagnosis. In addition, therapeutic interventions aiming at improvement of functional domains such as rehabilitation are unsuccessful in cancer patients in about one forth of published studies [25]. At baseline patients with and without history of cancer showed similar quality of life and physical capacity in our study, suggesting preselection of cancer patients when considering above literature. On the other hand, a contribution of cancer to baseline functional restrictions cannot be excluded since even in long-term cancer survivors symptoms like dyspnea and fatigue are commonly present [26]. Of note, patients with active cancer had a significantly worse symptom burden at baseline but showed a similar benefit of TMVR.

## Study limitations

The present study is a monocentric study including patients from a high-volume referral center for TMVR. However, when considering usual markers of morbidity such as age, EuroSCORE, and cardiovascular comorbidity, as well as long-term outcomes, our cohort is comparable with recently published real-world registries of patients with TMVR [2, 3, 8]. Larger prospective multicenter studies would strengthen the level of evidence for TMVR in cancer patients, but to the best of our knowledge the ongoing registries did not assess cancer history in detail.

Furthermore, despite a large study sample a subgroup analysis on individual cancer entities was not possible. Also, due to limited information on cancer therapies used in the long-term cancer survivors and the small patient numbers within respective treatment groups relations with TMVR outcomes could not be examined. Our study did not include cancer patients who underwent targeted tumor therapy for active cancer disease, for example with immune checkpoint inhibitors.

## Conclusion

The results of our study show that patients with cancer disease and symptomatic MR selected according to current guideline recommendations for TMVR show comparable clinical outcome with respect to midterm mortality and rehospitalization as non-cancer patients, albeit patients with active cancer had increased long-term mortality. Cancer patients showed similar improvement in exercise capacity, heart failure symptoms and health related quality of life. These results support the use of TMVR in patients with cancer but also highlight the importance of patient selection particularly if cancer disease is active.

### Supplementary Information

Below is the link to the electronic supplementary material.

Supplementary file1 (DOCX 500 kb)

## Data Availability

Not applicable.

## Abbreviations

TMVR: Transcatheter mitral valve repair
SF: Short Form
PCS: Physical component score
MCS: Mental component score
MLWHFQ: Minnesota Living with Heart Failure Questionnaire
NYHA: New York Heart Association
6MWT: Six-minute walk test
IQR: Interquartile range
MR: Mitral regurgitation
eGFR: Estimated glomerular filtration rate
