# Supplementary material for: Prognostic impact of cancer history in patients undergoing transcatheter mitral valve repair
Source: Clin Res Cardiol. 2023 Aug 15;113(1):94–106. doi: 10.1007/s00392-023-02266-5 (PMC10808190; doi:10.1007/s00392-023-02266-5)
Supplement: Supplementary file 1 — Supplementary file1 (DOCX 500 kb) [file 392_2023_2266_MOESM1_ESM.docx]

Suppl. Figure 1: Changes in functional parameters from baseline to 6 weeks in patients with cancer by history of chest irradiation


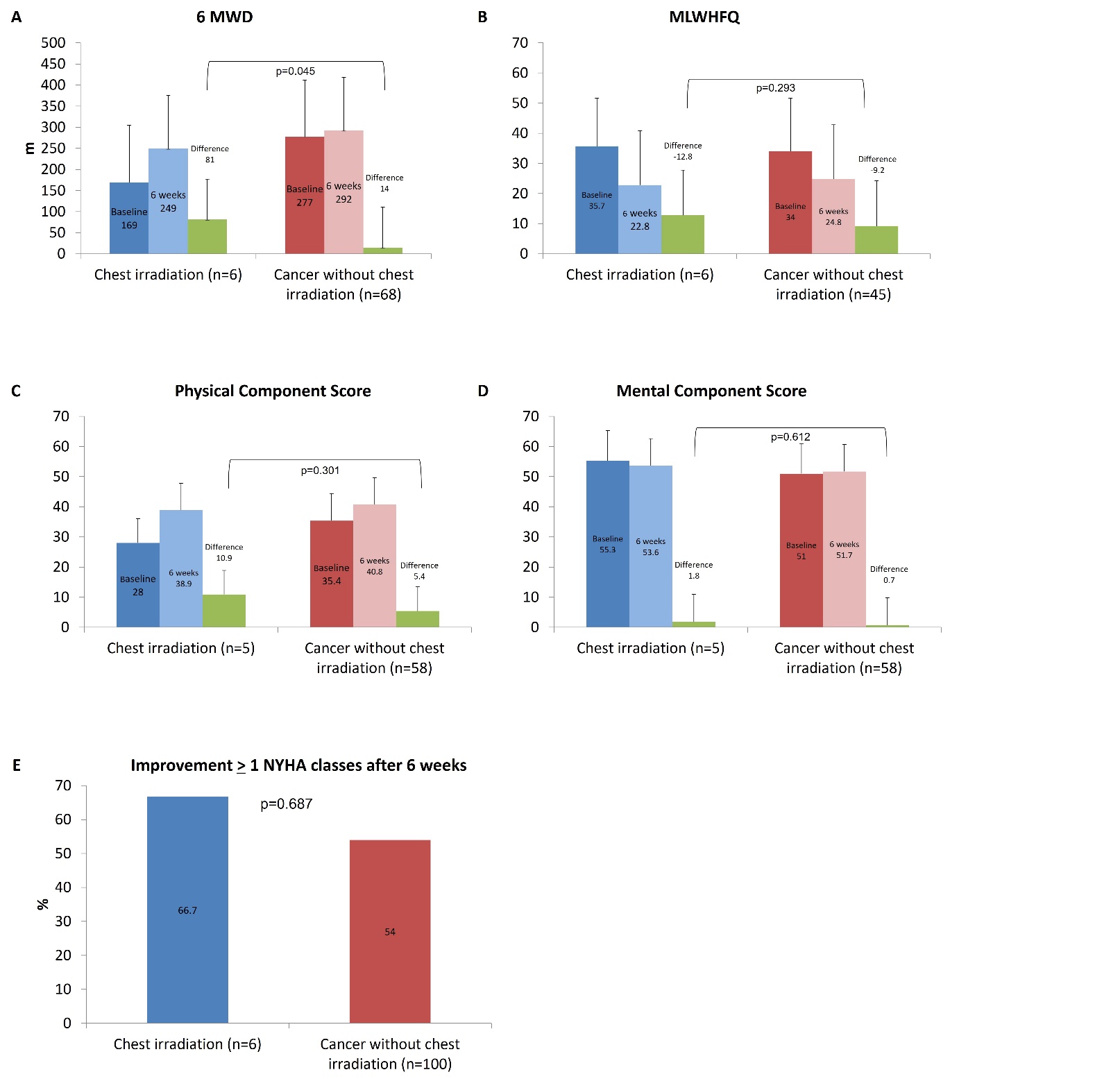


Depicted are baseline, 6-week, and absolute changes (delta) in 6 minute walking distance (6 MWD) (A), Minnesota Living with Heart Failure Questionnaire (MLWHFQ) score (range 0 to 105; higher scores indicate worse quality of life) (B), physical component (C) and mental component (D) scores (range 0 to 100; higher scores indicate better physical and mental status), and improvement of ≥1 New York Heart Association functional class at 6 weeks (E)

p-value for comparison of absolute changes between cancer patients with and without chest irradiation.

Suppl. Figure 2: Changes in functional parameters from baseline to 6 weeks in patients with cancer by presence of urogenital cancer


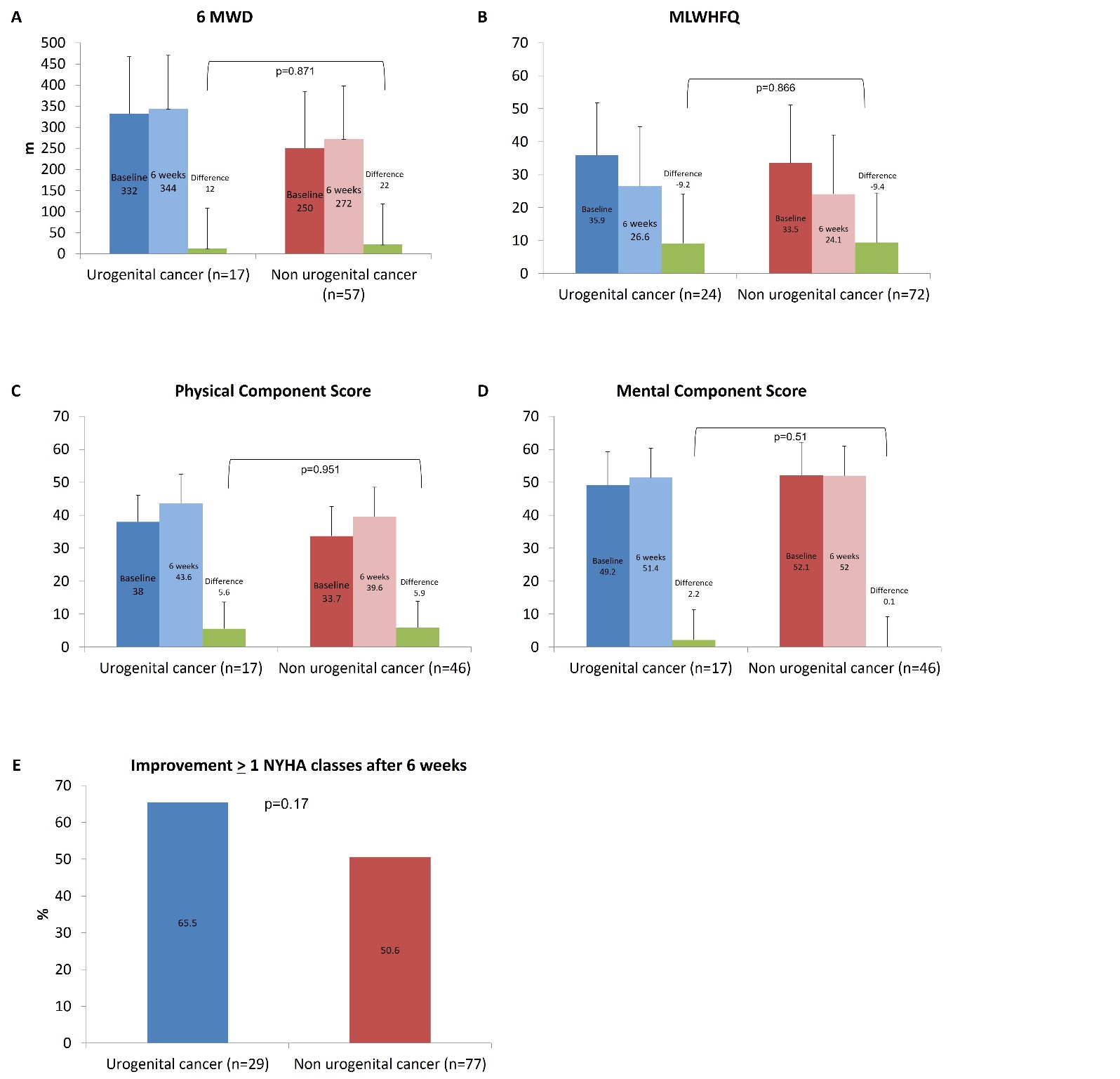


Depicted are baseline, 6-week, and absolute changes (delta) in 6 minute walking distance (6 MWD) (A), Minnesota Living with Heart Failure Questionnaire (MLWHFQ) score (range 0 to 105; higher scores indicate worse quality of life) (B), physical component (C) and mental component (D) scores (range 0 to 100; higher scores indicate better physical and mental status), and improvement of ≥1 New York Heart Association functional class at 6 weeks (E)

p-value for comparison of absolute changes between cancer patients with and without urogenital cancer.
